# The Vaccination Papers Approved

**Published:** 1898-06

**Authors:** Thomas M. Dillingham

**Affiliations:** New York


### THE VACCINATION PAPERS APPROVED.

NEW YORK, April 26th, 1898.

DEAR DR. JAMES:—By all means let us have all there is to say on vaccination, especially the papers of Dr. Leverson.

Yours truly,

THOMAS M. DILLINGHAM.

[Dr. Dillingham is the President of the International Hahnemannian Association.—ED.]

